# Barriers to tuberculosis case finding in primary and secondary health facilities in Ghana: perceptions, experiences and practices of healthcare workers

**DOI:** 10.1186/s12913-022-07711-1

**Published:** 2022-03-19

**Authors:** Joyce B. Der, Alison D. Grant, Daniel Grint, Clement T. Narh, Frank Bonsu, Virginia Bond

**Affiliations:** 1 TB Centre, London School of Hygiene & Tropical Medicine, London, UK; 2 Department of Epidemiology and Biostatistics, School of Public Health, University of Health and Allied Sciences, Hohoe, Ghana; 3 School of Laboratory Medicine & Medical Sciences, Africa Health Research Institute, College of Health Sciences, University of KwaZulu-Natal, Durban, South Africa; 4 School of Public Health, University of the Witwatersrand, Johannesburg, South Africa; 5 Department of Biostatistics and Informatics, Institute of Medical Biostatistics, Epidemiology and Informatics, University Medical Centre, Mainz, Germany; 6 Department of Disease Control and Prevention, National TB Control Program, Ghana Health Service, Accra, Ghana; 7 Department of Global Health and Development, Faculty of Public Health and Policy, London School of Hygiene and Tropical Medicine, London, UK

**Keywords:** TB, Case finding, Health facilities, Healthcare workers, Ghana

## Abstract

**Background:**

Ghana’s national tuberculosis (TB) prevalence survey conducted in 2013 showed higher than expected TB prevalence indicating that many people with TB were not being identified and treated. Responding to this, we assessed barriers to TB case finding from the perspective, experiences and practices of healthcare workers (HCWs) in rural and urban health facilities in the Volta region, Ghana.

**Methods:**

We conducted structured clinic observations and in-depth interviews with 12 HCWs (including five trained in TB case detection) in four rural health facilities and a municipal hospital. Interview transcripts and clinic observation data were manually organised, triangulated and analysed into health system-related and HCW-related barriers.

**Results:**

The key health system barriers identified included lack of TB diagnostic laboratories in rural health facilities and no standard referral system to the municipal hospital for further assessment and TB testing. In addition, missed opportunities for early diagnosis of TB were driven by suboptimal screening practices of HCWs whose application of the national standard operating procedures (SOP) for TB case detection was inconsistent. Further, infection prevention and control measures in health facilities were not implemented as recommended by the SOP. HCW-related barriers were mainly lack of training on case detection guidelines, fear of infection (exacerbated by lack of appropriate personal protective equipment [PPE]) and lack of motivation among HCWs for TB work. Solutions to these barriers suggested by HCWs included provision of at least one diagnostic facility in each sub-municipality, provision of transport subsidies to enable patients’ travel for testing, training of newly-recruited staff on case detection guidelines, and provision of appropriate PPE.

**Conclusion:**

TB case finding was undermined by few diagnostic facilities; inconsistent referral mechanisms; poor implementation, training and quality control of a screening tool and guidelines; and HCWs fearing infection and not being motivated. We recommend training for and quality monitoring of TB diagnosis and treatment with a focus on patient-centred care, an effective sputum transport system, provision of the TB symptom screening tool and consistent referral pathways from peripheral health facilities.

**Supplementary Information:**

The online version contains supplementary material available at 10.1186/s12913-022-07711-1.

## Background

Tuberculosis (TB) is a major global public health threat and in 2019, an estimated ten million people developed the disease with 1.2 million deaths among human immunodeficiency virus (HIV) negative people [1]. It was also estimated that 2.9 million TB cases were missed globally in the same year; this could be due to under-diagnosis, or under-reporting of detected cases. Under-diagnosis could arise from people with TB not accessing health care and/or being missed by the health system when they seek care [1]. Prompt diagnosis and treatment of TB is essential to prevent spread of infection.

Ghana is among the 30 TB and HIV high burden countries according to the World Health Organization (WHO) [2]. A national TB prevalence survey conducted in 2013 showed higher than expected TB prevalence. Among those identified to have TB in the survey, only 5% (9/202) had been diagnosed with TB before the survey and were on treatment during the survey period [3]. The national standard operating procedure (SOP) for TB case detection, developed in 2010, outlines what health facilities and healthcare workers (HCW) should practice to ensure clients with symptoms of TB who access care are identified promptly and initiated on treatment [4]. However, HCWs may encounter barriers that hinder their ability to detect people with TB promptly when they seek care from health facilities.

Qualitative studies from low- and middle-income countries have evaluated barriers to TB diagnosis and treatment in health facilities that can be classified as health system-related or patient-related barriers [5–9]. Several quantitative studies have assessed reasons for health systems delay in diagnosing TB, such as first seeking care from private health providers [10], health centres/clinics [11] or general practitioners [12] and multiple healthcare contacts [13, 14]. Qualitative studies in urban areas of Ghana have examined TB stigma and how it affects case finding [15, 16], however, no qualitative study has been conducted in rural primary health clinics to assess barriers to TB case finding in health facilities. Barriers to TB case finding in health facilities may vary across different contexts and understanding these barriers is critical to identifying potential interventions for improvement. This study was part of a larger project and qualitative research activities were conducted before and after quantitative components. Specifically, structured clinic observations were carried out before cohort and cross-sectional studies [17, 18] and in-depth interviews were carried out as the final research activity of the project. The quantitative studies showed gaps which were health system- and HCW-related. The aim of the qualitative enquiry in this paper was to assess barriers to TB case finding from the perspective, experiences and practices of HCWs and to explore their suggestions for sustainable ways to improve TB case finding in health facilities in Ghana.

## Methods

### Study setting

The study was conducted in the southern part of the Volta region, Ghana, in a municipality that has one of the highest burdens of TB in the region [19]. In 2018, the municipality notified 172 TB cases out of a target of 546 estimated based on the 2013 national TB prevalence survey [20]. For the study, from the municipality’s eight rural health facilities, we selected four which were geographically spread and had the highest outpatient department (OPD) attendance, and the municipal hospital, where the only TB diagnostic laboratory and chest clinic were based.

In every health facility, the national SOP for TB case detection states that there should be a TB team made up of facility stakeholders that is responsible for planning, monitoring and evaluating adherence to TB case detection protocols. The regional and district TB teams are supposed to visit health facilities quarterly to monitor case detection activities. The SOP also indicates that all clients attending OPDs of all health facilities should be asked about cough, irrespective of presenting symptoms. Those who report a cough should be screened with a TB symptom questionnaire (a screening tool) [4]. Clients who report a cough of two weeks or more, or clients with cough of any duration plus two or more TB-related symptoms (fever, chest pain, weight loss and night sweats), should be asked to do a sputum TB test, and be recorded into a cough register. Also health talks on the basics of TB and TB infection control should be given to groups of clients at the OPD. These health talks are normally given in the morning at the client OPD waiting area.

### Study design and population

Two qualitative methods were used: structured clinic observations and in-depth interviews. The eligibility criteria for clinic observation were a space and process of relevance to TB client flow and that for in-depth interviews were HCWs involved with TB case finding in the selected health facilities. The participants for in-depth interviews were purposefully chosen and included one medical doctor, two physician assistants, eight nurses and one task-shifting officer. There were four women and eight men participants. Their ages ranged from 27–42 years. Four of the participants were from rural health facilities. The number of years of experience in TB work ranged from six months to three years.

### Data collection and procedure

#### Clinic observations

Clinic observations were conducted in May 2018. The observations were done by JBD and CTN at the OPD waiting area, triage area and consulting rooms of the study health facilities and the laboratory and chest clinic of the municipal hospital. Observations were done using an activity observation report form and a checklist [21] that included: waiting time of clients at the OPD waiting area; procedures at the triage area; interaction between HCW and clients in the consulting room; waiting time and dropping samples at the laboratory; and procedures at the chest clinic (Additional file 1). Notes were taken during the observations and filled out in more detail immediately after the observations were completed. These observations were done between 7am and 12pm on days that the health facilities were very busy (usually market days). Two days were spent at each facility and on each of the days, two researchers followed client flow by observing clients as they moved from one area to the other. The researchers dressed casually and were introduced to HCWs at the facilities before they started the observations.

Based on an initial assessment of health facilities in April 2018 before the start of data collection, we found that there were no TB case finding activities in the rural health facilities. This made it necessary to introduce the screening tool to HCWs who attended to patients in the consulting rooms of the study rural health facilities to facilitate enrolment for the cohort study [18] and also as an ethical obligation to help improve TB services. The HCWs were trained by the research team on how to use the screening tool and printed copies of the tool were provided to the rural health facilities. This was done just after the clinic observations at the beginning of May 2018. The quantitative studies were then conducted from May to December 2018 followed by the in-depth interviews in January 2019.

#### In-depth interviews

In-depth interviews were held in the health facilities at a time that was convenient for the HCW and lasted on average 50 min. Some of the HCWs in the rural health facilities who had been trained to use the TB symptom screening tool were among those interviewed. Interviews were led by JBD, a female PhD student from Ghana trained in qualitative research who has worked as a TB laboratory focal person in one of the regions in Ghana; and a trained research assistant observed and took notes. All interviews were conducted in English because this is the official language used by HCWs for professional interaction. Interviews were audio recorded. An interview guide based on findings from clinic observations was used and included training in TB control, practices in TB diagnosis and treatment, experiences in TB case finding, barriers to TB diagnosis and treatment and solutions suggested by the HCWs (Additional file 2). The interview guide was piloted in a health facility different from the study health facilities and some questions were revised for clarity. Prior to the in-depth interviews being conducted, JBD had been living in the municipality for seven months collecting data on other components of the study in the selected health facilities and had developed rapport with the HCWs.

### Data analysis

#### Clinic observations

Immediately after the clinic observations, data were entered in Excel spreadsheets by JBD with columns representing observations done per area of the health facility and rows representing each health facility. Data were organized by the type of health facility. Patterns across the different areas of the health facilities observed could then be identified. Narrative data based on the observed patterns were used to show the workflow and practices of HCWs when clients reported to the health facility, for example, practices at the triage area, consulting room, laboratory and chest clinic. The number of patients screened for TB symptoms, number reporting a cough and number asked to submit a sputum for TB test were noted during observations and computed. This allowed the observations from the facilities to be used to validate the findings from the in-depth interviews and to thus triangulate, enrich and deepen the understanding of the data.

#### In-depth interviews

All audio recordings were transcribed by JBD. Thematic analysis was employed where JBD first familiarized herself with the data by reviewing the transcripts while listening to the recordings. This was also done as a quality control step. The transcripts were also reviewed by VB who was the qualitive expert on the study. The transcripts and field notes were then summarized and read over to identify emerging themes. JBD and VB then discussed and agreed on the themes. Summaries from clinic observations and transcripts were triangulated to generate the final analytical themes. Themes were organized in a framework that categorized barriers into two main areas: (i) health system-related factors, which are factors relating to the operations of the health system; and (ii) HCW-related factors relating to attitudes of HCWs. These categories were adopted from models used by Cattamanchi et al. and Chimbatata et al. [5, 22].

### Ethical considerations

Ethical approval for the study was obtained from the Ghana Health Service Ethics Review Committee (GHS-ERC:003/09/17) and London School of Hygiene & Tropical Medicine Ethics Committee (LSHTM Ethics Ref:14504). Written informed consent was obtained from all HCWs who participated in the in-depth interviews. HCWs who were present during clinic observations were informed of the purpose of the observation and given the opportunity to ask questions before commencement.

## Results

We first present the TB client flow and standard TB management in the two types of health facilities, before presenting findings about barriers that were categorized as health system-related and HCW-related barriers. A table of illustrative quotes (Additional file 3) is presented as supplementary material.

### TB client flow and standard TB management in health facilities

**Fig. 1:**
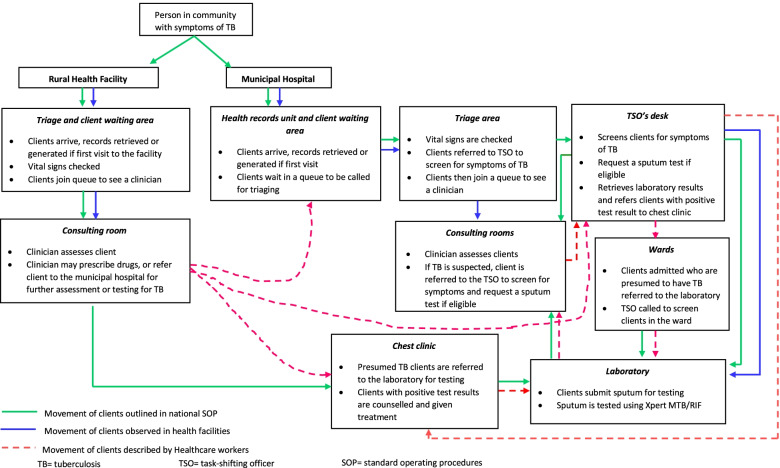
TB client flow and management in rural health facilities and the municipal hospital

Figure 1 shows the TB client flow and actions that were taken at each step of the pathway in rural health facilities and the municipal hospital. Client flow in all facilities was similar until after triaging where, in the municipal hospital, clients first saw a task-shifting officer before queuing to see a clinician. The task-shifting officer was a HCW designated to screen for symptoms of TB and request sputum tests.

### Rural health facilities: Health system-related barriers

#### Lack of diagnostic facilities in rural health facilities

In the study setting, the only TB diagnostic facility was in the municipal hospital. Therefore, HCWs in rural health facilities had to refer all presumed TB clients to the municipal hospital for further assessment and testing. However, HCWs in all the four rural health facilities complained that most clients who were referred never reached the municipal hospital. In addition, the referral system for presumed TB clients varied. Two facilities gave clients a referral letter to the chest clinic at the municipal hospital, one facility referred clients to the task-shifting officer and the other facility referred clients to the general OPD at the municipal hospital. Study observations documented that clients who were referred to either the chest clinic or the task-shifting officer were attended to more quickly than those referred to the general OPD.

One HCW said at times they needed to threaten clients to go to the municipal hospital for TB testing.

“I have experienced that, most of them don’t go [….] unless we tell them that maybe they will die before they will just rush to go and come”. (Male HCW, Rural health facility [RHF]).

HCWs said extended waiting times and being treated badly by staff at the municipal hospital was off putting for clients they referred. Indeed, some HCWs relayed how consequently some clients would suppress their cough or would not report their TB-related symptoms to the HCW because they did not want to be referred to the municipal hospital, and that in extreme cases people may die because they did not go to hospital as recommended.

“[…] sometimes patients want to suppress the symptoms because they know you will refer them and if you refer them, they won’t go because of financial constraints. So, when you start going deeper, they tend to refrain from answering you more and that way we don’t get to capture the whole history about the patient. A patient could be coughing like two weeks and will come and tell you it started three days ago […]”. (Female HCW, RHF).

One reason for not even referring clients for a TB test was HCWs assuming that their clients could not afford the cost of transport to the municipal hospital for further assessment and/or to do a TB test. The rural facilities were about 10 to 20 km from the municipal hospital.

“[…] if they hear the name X (municipal hospital) they will start crying because they don’t have money for T&T (travel and transportation)”. (Female HCW, RHF).

Another reason for not referring clients was related to fishing communities. During the peak of the fishing season, most fisherfolk migrate to neighbouring countries where they work on big fishing vessels for better income and only return after several months. Therefore, HCWs were reluctant to refer such clients during the fishing season, assuming they would opt for their livelihood over the referral.

“Some of them you will not see them again after you refer them […] they will travel to Benin, Togo or Ivory Coast. Those are the places they have been going because of the fishing, they will go to fish there and it makes follow up difficult”. (Female HCW, RHF).

#### Suboptimal screening for TB symptoms and sputum test request

Clinic observations showed that in three of the four rural health facilities, HCWs at the triage area were not asking clients about cough. In the consulting rooms, clients were asked about cough as part of a routine set of symptoms asked to all clients. However, clients who reported a cough were not screened with the TB symptom screening tool to determine their eligibility for a sputum test. Of 78 clients observed across the four rural health facilities in May 2018, 27 (34.5%) reported a cough to a HCW in the consulting room but none of them was screened with the screening tool. HCWs said they had never seen the screening tool until the study team introduced it to them. During initial clinic observations, the screening tool was not found in any of the consulting rooms of the health facilities.

“I think the first time we had it (screening tool) was when you provided it, to be very frank. We never had any guideline (screening tool) for TB until you provided the TB guideline (screening tool)”. (Female HCW, RHF).

During HCW interviews conducted in January 2019, after the symptom screening tool had been introduced, some of them said all clients in the consulting room were asked about cough and that the screening tool was used when appropriate. However, no observations were conducted after to triangulate whether this change in practice had been made. Other HCWs shared that they did not ask all clients about a cough.

“A patient could come with hypertension, we don’t ask for cough so I won’t say all patients, but I will say majority of patients, especially with the malaria cases […] but not all patients”. (Female HCW, RHF).

Asked why HCWs were not asking about cough or using the screening tool, HCWs responded that there was shortage of staff leading to heavy workload.

“We don’t have enough staff [..] Actually the one who was doing this is no longer in the facility”. (Female HCW, RHF).

#### Sub-optimal infection prevention and control practices

In all the rural health facilities, we observed that health talks on TB and other health conditions were not given at the clients’ waiting area. When we interviewed HCWs, they said health talks were provided on one-on-one basis with clients who reported a cough and they normally advised them to cover their mouths when they are coughing. Again, in all health facilities, we observed that clients in the waiting area who were visibly coughing were not isolated from the crowd and attended to, as required by the national SOP for case detection. In one rural health facility, the HCW admitted that they did not have enough space to isolate clients with cough. Further, fast-tracking them through the process created misunderstanding with other clients already in the queue.

“You only tell them to use their handkerchief to cover their mouth when they are coughing because this place is just too small so you can’t be isolating […] and sometimes too some came to meet others so if they are overpassing it will bring confusion”. (Female HCW, RHF).

#### Insufficient monitoring and supervision of TB work

None of the rural health facilities had a TB team or focal person to ensure HCWs were following protocols. Also, there was poor documentation in cough registers at all the rural health facilities, and no supervision of TB case finding activities. The last record of cough was entered more than a year ago prior to this research. HCWs explained this as due to “laziness”, not enough staff to do entries, heavy workloads and inadequate registers.

“You see this place that we are working, the workload is too much for us, because you are the only person consulting, dispensing at the same time dressing wound and other things. So, most times doing recordings in the book is difficult. So if you take one client and you do all these kind of things by the time you realize the day is over”. (Male HCW, RHF).

### Healthcare worker-related barriers

#### Gaps in TB knowledge and lack of training in case detection guidelines

HCWs believed some of their colleagues might have less knowledge about TB, especially the signs and symptoms. Therefore, when they examine clients with symptoms of TB, they do not think of TB but rather other health conditions. To compound this problem, the majority (75%) of the HCWs interviewed had received no training on TB and had no idea about the national SOP for TB case detection. Indeed, at the start of this study, the SOP was not available in any of the health facilities. HCWs believed the lack of training on the SOP led to majority of them not knowing what to do when they see a client with cough or TB-related symptoms. They believed that training should be done regularly because of the high turnover of staff.

In addition, the lack of training on the SOP for case detection led to different criteria being used by different rural health facilities to refer a client to the municipal hospital for further assessment or for a TB test. In one facility, cough ≥ 2 weeks was used as criterion for referring clients for a TB test, in another facility, cough and weight loss were used. In the two other facilities, they used cough and other TB-related symptoms. A respondent at one rural health facility felt lack of training should not prevent HCWs from identifying people with symptoms of TB.

### Municipal Hospital: Health system-related barriers

#### Suboptimal screening for TB symptoms and sputum test request

At the municipal hospital, clients were supposed to be asked about cough during triaging. Those who reported a cough were to be referred to the task-shifting officer to be screened for TB-related symptoms and, if eligible, be asked to submit sputum for a TB test. However, clinic observations showed that after being triaged, patients were not sent to the task-shifting officer to be screened for TB-related symptoms. Rather, the task-shifting officer either actively identified clients who were coughing in the waiting area and approached them to be screened and had a sputum test requested if they were eligible, or clients identified in consulting rooms of the municipal hospital who required screening were referred to the task-shifting officer.

Some HCWs said the reasons they were not asking about cough or using the screening tool were that it was not their job, that it was extra work and at times they forgot to ask about cough.

“They are complaining that it’s not their job and they sometimes forget to ask. They cannot be checking the vital signs and at the same time remember to be asking about cough”. (Male HCW, Municipal hospital [MH]).

All the clinicians at the municipal hospital said they had never seen the screening tool or sputum test request forms in the consulting room. However, HCWs at the chest clinic disagreed with their colleagues saying all HCWs were trained on how to use the screening tool and all forms needed were distributed to all the departments but they refused to use them.

“They don’t ask about cough, but they are supposed to ask the clients, but they are not doing it, meanwhile they’ve been trained but yet still they are not doing it. Apart from the task- shifting officer asking about cough, the screening tool was sent to every unit, consulting rooms but now they are not doing it at all, […] now everything is on the task-shifting officer and the TB unit”. (Male HCW, MH).

Many HCWs in the municipal hospital alluded that TB diagnosis would be more efficient if they were also screening for symptoms of TB and requesting for sputum test directly instead of referring to the task-shifting officer.

#### Sub-optimal infection prevention and control practices

HCWs from the chest clinic complained that when they tried to let clients who were coughing see the doctor quickly to prevent potential spread of infection, they faced opposition from their colleague HCWs at the OPD. Also, entry to the isolation wards for TB clients was not restricted and the time spent in the ward by client relatives was not regulated.

“[…] mostly I try my best to let them see the doctor fast but sometimes it brings some kind of argument between I and the nurses because they will say they can’t allow me to jump the person over other people in the queue […], this person just came some few minutes ago […]”. (Male HCW, MH).

In the municipal hospital, there was a facility TB team in theory, but it was described by some key HCWs as non-functional for the past two years. HCWs with TB responsibilities felt that the ineffectiveness of the team was what was causing other HCWs not to adhere to the SOP for TB case detection.

“Previous minutes from the files show the team used to meet […] and discuss the challenges and find some ways of resolving them but since I joined the team in 2016, frankly speaking the team is not working well, is not working, it was working previously but now is not working since they had different administration [….], it is no more effective again”. (Male HCW, MH).

### Healthcare worker-related barriers

#### Fear of infection and attitude towards TB work

Fear of infection was one of the main barriers affecting TB case detection in the municipal hospital. Nurses in the wards were reluctant to attend to TB clients admitted to the isolation wards of the hospital because they were worried about getting infected. This fear also prevented other HCWs from being interested in TB work. The fear was attributed to the lack of essential personal protective equipment (PPE) like N95 respirators. HCWs claimed the hospital management said N95 respirators were expensive and not readily available.

“So everybody is on the alert, I don’t want to be infected, I don’t want to get infected and that thing has brought in some reluctancy in getting closer to TB clients or TB unit […]” (Male HCW, MH).

HCWs at the OPD mentioned that because of the constant complaints by other HCWs who are supposed to attend to TB patients in the isolation ward about lack of PPE, this had influenced some OPD staff to refuse to see clients with productive cough or with TB.

“[…] so, the ones who are not coughing I mean we can do our normal examination but most of the time I mean we don’t wear any PPEs. There have been few times which I have declined to see TB patients or those with productive cough […]”. (Male HCW, MH).

HCWs at the chest clinic said the presence of a task-shifting officer, coupled with lack of motivation, had undermined the interest of other HCWs in TB work. Perceived incentives for the task-shifting officer and other staff of the chest clinic from the national TB control programme reinforced that they should be the only ones screening for symptoms of TB and requesting for sputum test. It was reported that at times that the task-shifting officer was not available, for instance on sick leave, no one did his job. They also said this responsibility shifting could lead to delay in diagnosis and more inconvenience and cost for presumed TB clients.

“[…] I’ve heard statements whereby somebody is coughing, and they will say they should call the task-shifting officer because he is being paid to do that work, that one I’ve heard it several times”. (Male HCW, MH).

In addition to the health system and HCW-related barriers, HCWs perceived some patient-related barriers that were similar for both the municipal hospital and rural health facilities. These barriers are outlined below:

### Gaps in knowledge of TB

Most HCWs in all the health facilities relayed that TB knowledge among people in the communities was limited. They claimed that most community members did not know about TB as a disease or the signs and symptoms of TB, and would usually not associate cough with TB. They said that community members would buy cough syrups from the drug stores or take herbal medicines, leading to a delay in seeking care. According to HCWs, some community members regard TB as a death sentence, leading to despondency towards treatment and recovery.

### Traditional beliefs

HCWs referred to traditional and/or spiritual beliefs in the study area that associated TB with punishment for doing something wrong, especially infidelity. Therefore, when people develop severe cough, they seek help from traditional shrines or prayer camps, delaying attending the health facility until their health condition has worsened. One clinician reported that because of alternative beliefs, some people who start TB treatment will throw the medication away and resort to herbal treatment.

“They have been in a specific culture; specific tradition and they think that tradition is helping them. You will give somebody treatment he will throw the medicine away, go in for some herbal medication thinking that is good […]”. (Male clinician, MH).

### Stigma

TB related stigma was reported by HCWs to lead to community members hiding TB symptoms instead of seeking care from the health facility.

“Sometimes the level of stigmatization in the community is very high. Sometimes most of them they don’t want their relatives and their loved ones and their friends to know that they have the condition so they may be hiding unless we are able to get them, talk to them and bring them in for treatment”. (Male HCW, MH).

Further, community members associate TB with HIV and this contributes to delays in seeking care and/or not accepting a TB diagnosis and refusing treatment. HCWs at the municipal hospital said it was common to see community members who were more educated and/or had a high social status refuse to accept a TB diagnosis because of the stigma associated with the disease. 

**Fig. 2:**
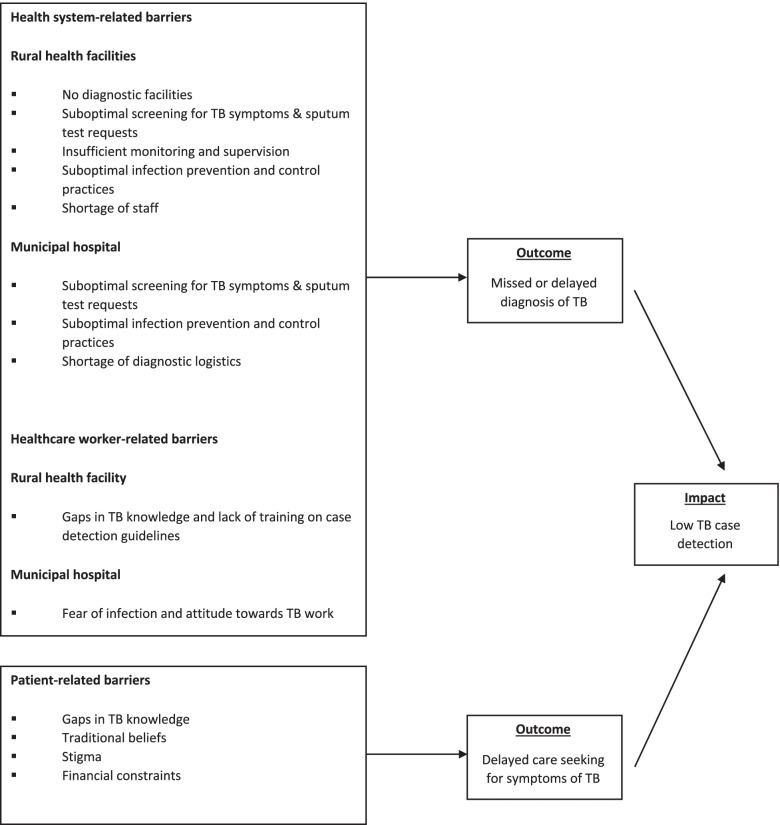
Summary of barriers to TB case detection in health facilities in southern Volta region Ghana

A summary of barriers to TB case detection in health facilities is shown in Fig. 2. The diagram shows that health system-related and healthcare-worker related barriers lead to missed or delayed diagnosis of TB and the impact of these is low TB case detection.

### Solutions suggested by healthcare workers

In rural health facilities, some HCWs mentioned that if there could be a diagnostic facility in each sub-municipality, then clients would not have to travel to the municipal hospital for the TB test. Other HCWs thought that laboratory personnel going round the rural facilities and collecting sputum specimen from presumed TB clients would also help to reduce the loss-to-follow up due to referrals to the municipal hospital.

Most of the HCWs in both rural and urban facilities mentioned sensitization and training of HCWs on the SOP for TB case detection. They recommended that training should be routinely held because of the high turnover of staff. Providing logistics such as N95 respirators to ensure HCWs feel safe in dealing with clients with cough or TB were prioritized.

HCWs felt that there should be a functional TB team or focal person in every health facility to monitor TB case detection activities and to ensure HCWs adhere to the guidelines. Apart from a functional health facility TB team, there should be regular monitoring and supervisory visits from the national, regional and district TB teams to ensure that guidelines are followed and also to serve as motivation for the HCWs to know that their work is appreciated. Provision of funds to health facilities to support patients with their transportation cost to the municipal hospital would be helpful.

The main themes, sub-themes, summary of context specific key findings and proposed solutions to barriers identified can be found in Table 1.

**Table 1:** Themes, summary of key findings and proposed solutions to barriers identified

Main theme	Sub-theme	Summary of key findings	Proposed solutions
**Health system-related barriers**	Lack of diagnostic facilities in rural health	Contextual barriers: specific to rural health facilities • Led to referral of presumed TB clients to the municipal hospital 10–20 km away for sputum testing, involving time away from livelihoods (e.g. fishing) and transport cost • Differences in referral processes, with referral to chest clinic and/or task-shifting officer more efficient than referral to OPD	• Transport subsidies • Training to make referral processes consistent
	Suboptimal screening for TB symptoms and sputum test request	Contextual barriers: specific to rural health facilities • Clients reporting cough were not screened with the TB symptom screening tool • TB symptom screening tool not available Contextual barriers: specific to municipal hospital • Clients not asked about cough at the OPD and in the consulting rooms and TB symptom screening tool not used because HCWs felt it was not their job • Only the task shifting officer asked about cough and used the screening tool	• Training on SOP for TB case detection • Provision of screening tool to rural facilities • Proper integration of task shifting concept into routine OPD care
	Sub-optimal infection prevention and control practices	Contextual barriers: rural health facilities and municipal hospital • Clients visibly coughing at the waiting area not isolated and attended to (e.g. due to lack of space in rural health facilities) • Clients reporting a cough were not fast-tracked through the process of seeing a doctor because it created misunderstanding with other clients already in the queue or HCWs at the OPD	• Structural provisions (e.g. well-ventilated space or room) should be made for isolation of clients who visibly cough at the waiting area • Education of clients and HCWs on the need to fast-track clients visibly coughing through the process at the OPD
	Insufficient monitoring and supervision of TB work	Contextual barriers: specific to rural health facilities • No TB team or TB focal person to ensure SOP on case detection was followed • Poor documentation in TB registers Contextual barriers: specific to municipal hospital • Non-functional TB team which led to poor supervision of TB case detection activities	• Formation of TB teams or appointment of TB focal persons to monitor adherence to SOP for TB case detection • Reactivation of the TB team at the municipal hospital to supervise TB case detection activities and ensure adherence to guidelines
**Healthcare worker-related barriers**	Gaps in TB knowledge and lack of training in case detection guidelines	Contextual barriers: specific to rural health facilities • Majority of HCWs had not received training on TB case detection and had no idea about the SOP for case detection which led to missed opportunities for early detection of persons with TB • SOP on case detection not present in any of the facilities • Reported bad treatment by municipal hospital staff put clients off • Clients reportedly suppress symptoms to avoid referral	• Training on SOP for TB case detection • Providing the SOP to all health facilities for reference • Training to address staff attitude to TB services and clients • Health education to facilitate clients being open about symptoms
	Fear of infection and attitude towards TB work	Contextual barriers: specific to municipal hospital • HCWs reluctant to attend to TB clients in isolation ward due to lack of PPE (e.g. N95 respirators). They feared they would get infected and would not be well compensated • This attitude led to HCWs at the OPD not also being interested in attending to clients presumed to have TB for the same reasons • The fear of infection could possibly lead to stigmatization of TB patients or clients with cough • HCWs felt it was the duty of the task shifting officer and staff of the chest clinic to attend to TB and presumed TB clients with the assumption that they received incentives for TB work	• Training to address staff attitude towards TB or presumed TB clients • Sensitizing HCWs on the need for collaboration between all units in the hospital to improve TB services • Hospital management should provide the appropriate PPE for TB work to alleviate fear of infection
**Patient-related barriers**	Gaps in TB knowledge	• Poor knowledge on signs and symptoms of TB led to patients buying cough syrups and herbal medicines • Led to some patients not seeking care from health facilities • Led to refusal by some patients reporting a cough to be screened for TB	• Education and sensitization of community members through community durbars (gatherings in the community organized by community leaders) as well as local radio stations on the signs and symptoms of TB and the need to seek care early from the health facilities
	Traditional beliefs	• Beliefs that TB was a spiritual disease or a punishment from the gods for wrong doing • Led to people seeking care from shrines or prayer camps which worsened their symptoms	
	Stigma	• TB associated stigma in the community led to people hiding their symptoms and not seeking care from health facilities • Association of TB with HIV led to people not seeking care and some people of high social status refused to accept TB diagnosis and treatment	

*TB* tuberculosis, *HIV* Human immunodeficiency syndrome, *OPD* outpatient department, *HCW* healthcare worker, *SOP* standard operating procedures, *PPE* personal protective equipment

## Discussion

In this study we found there were barriers to TB case detection, which are presented using a framework of health system-related and HCW-related barriers.

The main health system-related barrier was that the TB diagnostic laboratory was at the municipal hospital which is distant from most rural health facilities, and clients cannot afford the transport cost [18]. Limited access to diagnostic facilities, mostly in rural settings, and long travel distance were similar barriers experienced by presumed TB clients in other parts of the world [6, 7, 22–26]. The lack of a standard referral system in our rural study facilities also led to extended waiting times for some referred clients at the municipal hospital and this further underscored their reluctance to go to the municipal hospital for further assessment and testing.

Suboptimal TB screening practices found in all facilities may result in missed opportunities for early identification of clients with TB that could further impact on the spread of TB in the community. The lack of training and absence of the screening tool that we observed in this study could account for the HCWs not asking clients about cough as required by the national SOP for case detection. Harper et al. in their study in The Gambia also revealed through observations that HCWs did not adhere to the stated health policy on asking about cough and referral of presumed TB patients to the national TB control program (NTP) for further assessment [23]. Similarly, in China, Xu et al. found that HCWs were not alert to symptoms of TB and did not screen for other TB-related symptoms or request for a sputum test [27]. The “Stop TB partnership’s” “Action framework for higher and earlier TB case detection” recommends training of all HCWs, and not just HCWs in the TB unit, to ensure comprehensive implementation of existing diagnostic algorithms in health facilities to improve TB case detection [28]. Both this study and other research identified HCWs not being appropriately trained as a barrier to TB case detection [7, 22]. Shortage of staff and heavy workload were mentioned by HCWs as explanations for this non-adherence to TB case detection SOP, similar to other studies [5, 22, 29, 30].

The absence of a dedicated health facility TB team could be one of the reasons for the non-adherence to the SOP. We found insufficient monitoring and supervision of TB work in the health facilities because there were no TB teams or focal persons in any of the rural health facilities and the one in the municipal hospital was described as non-functional. HCWs in the rural health facilities and those in the chest clinic of the municipal hospital perceived that if there was a team regularly supervising and monitoring the activities of HCWs then that would prompt them to ask about cough and request for sputum test where necessary. Lisboa et al. in their study in Mozambique found that the lack of a motivated TB taskforce to supervise and monitor TB control activities in the health facility was a potential factor contributing to poor quality TB care [26]. In addition, HCWs in our study were of the view that if TB teams from the district, regional or national levels regularly visited the health facilities to monitor and encourage staff, this appreciation of their efforts combined with knowing their activities are going to be monitored would serve as a motivation for them to pay attention to TB case detection. HCWs in another region in Ghana expressed similar sentiments [31]. Moreover, in our study, HCWs were not putting into practice the recommended infection control measures which could lead to transmission of infection within the health facility. Comparably, in Nigeria, Tobin-West et al. found poor TB infection control practices in both rural and urban health facilities [32].

The main HCW-related barrier identified in this study was fear of infection that affected attitudes to TB work and undermined effective screening for symptoms of TB. A study from Malawi in 2015 also reported that fear of infection by HCW led to underassessment of clients for symptoms of TB [5]. The lack of PPE for HCWs compounds this fear. This same challenge was reported by Lisboa et al. in Mozambique and Dordor et al. in Ghana [26, 31]. This fear of infection was linked to stigmatization of TB or presumed TB clients as reported in another study in the Western region of Ghana [15]. In some rural health facilities, clients who were coughing at the OPD could not be isolated due to lack of space. This creates a complex barrier because the SOP states that it has to be done but no provisions are made. There is the need to make structural provisions (e.g. well-ventilated space or room) for isolation of patients who are visibly coughing at the OPD. In the municipal hospital, the lack of interest in TB work led to all the work being left to the task-shifting officer and HCWs at the chest clinic. This could lead to clients with symptoms of TB going through the health system without being identified by other categories of staff. Contrarily, in the Mozambican study, HCWs were advocating for task shifting in TB work where auxiliary staff can be trained to screen for symptoms of TB and believed that this would solve the problem of TB case detection [26]. In addition, HCWs’ perceptions that some incentives were given to the task-shifting officer and chest clinic staff meant that they were not responsible to screen for symptoms of TB and request for sputum test. These findings were consistent with findings from other parts of Ghana where perceived incentives for frontline TB staff and lack of interest in TB work by clinicians were identified as barriers to TB control [33]. The words people use can provide clues about the quality of their relationships. HCWs in the chest clinic and the task-shifting officer in this study often used words that implied other HCWs were not cooperative concerning TB case finding in the hospital. This suggests strained relationships between hospital staff which could further undermine TB screening in the facility. Moreover, TB infection prevention and control measures are needed to alleviate HCWs fear of infection when dealing with presumed or TB clients. Addressing HCWs concern about their risk of TB acquisition could improve TB case detection. The use of masks is more relevant in this era of the COVID-19 pandemic as they have been shown to reduce both transmission of and acquisition of SARS-CoV2 [34]. In addition, HCWs need to be educated on the fact that TB patients on treatment and admitted in wards are unlikely to be infectious especially after two weeks of anti-TB treatment [35] but rather it is the unidentified, untreated TB patient who is most likely to be infectious.

Aside from barriers linked to the health system or HCWs themselves, HCWs perceived some patient-related barriers that hinder TB case detection. Gaps in TB knowledge were a main barrier mentioned in this study. Misconstrued knowledge about TB was linked to traditional beliefs and lack of TB education in communities. One traditional belief linking TB to infidelity was also found by Chimbatata *et al.*in their study in northern Malawi [5]. HCWs in our study believed this gap in knowledge led to delay in seeking care. Other studies among HCWs have identified poor TB knowledge leading to delayed health care seeking as a barrier in TB control [27, 36]. Stigma was another barrier that reportedly prevented people from seeking care from health facilities. TB is normally associated with HIV in the community so some people with symptoms of TB fear to go to the hospital because they might be told they have HIV and delaying care leads to worse symptoms. This was comparable to findings from Zambia [37].

Many health care organizations have embraced patient-centred care as central to their strategic missions and values [38]. Patient-centred care involves providing care that is compassionate, empathetic, and responsive to the needs, values, and expressed preferences of each individual patient [39]. The care providers must understand the patient’s context and provide services to meet their needs. Barriers to TB case finding in health facilities are complex and innovative solutions should be patient-centred. In rural health facilities, HCWs suggested presumed TB patients should be paid to travel to the municipal hospital for a sputum test. This is a patient-centred approach that could be considered by the NTP reflecting on feasibility and sustainability of such an approach. In a randomized control trial in the Philippines on the effects of incentives and subsidies on TB testing rates in rural settings, participants who were provided with transportation and food costs had a higher odds of attending a health facility for TB testing [40] and in Uganda, participants who were given transportation vouchers or cash transfers were willing to return to a health facility to complete testing [41]. Another patient-centred measure that has worked in other settings is the implementation of a specimen transport system where presumptive TB patients are asked to produce sputum at the rural health facility. The sputum specimen is then transported to the diagnostic laboratory for testing instead of referring patients to travel to the diagnostic laboratory to submit sputum. In South Africa, Naidoo et al. found only 5% of TB patients could not access testing [42]. Naidoo et al. suggested that the effective sputum specimen transport system in South Africa could have accounted for the low proportion of patients not accessing testing. Ghana’s NTP can adopt such patient-centred strategies to improve TB case finding in rural facilities. Also, implementation of a standard patient referral system between rural health facilities and the municipal hospital, which was lacking in the study setting might improve TB case finding.

Our study has potential limitations. HCWs might have been more comfortable highlighting some barriers than others. The dominance of one health professional category (nurses) in the study population might affect the diversity of the information obtained. The choice of market days for clinic observations could have introduced some bias since these days were busier and so it was possible that HCWs had less time to spend with each patient and therefore could have been less thorough. The process of observation may have changed HCWs behaviour, but would be expected to promoted adherence to guidelines. Thus what was observed is likely to be closer to guidelines than “normal” unobserved practice. Also, we did not interview TB patients for their perspective on barriers to TB diagnosis and treatment. However, the strength of this study was the use of both interviews and health facility observations to validate our findings.

## Conclusion

In southern Volta region, Ghana, the main health system barriers to TB case detection reported by HCWs were lack of TB diagnostic laboratories in rural health facilities, fear of infection and suboptimal adherence to case detection protocols. These barriers likely contribute to poor TB case detection rates in the municipality. The barriers identified portray a complicated health system with no “one size fits all” solution. There is a need for appropriate interventions that focus on patient-centred care such as to improve TB symptom screening, an effective sputum transport system and standard referral linkage to bridge the gap between rural health facilities and laboratories so that people with TB are not lost even before diagnosis.

## Supplementary Information

**Additional file 1.**

**Additional file 2.**

**Additional file 3.**

## Data Availability

The datasets (interview transcripts and clinic observation notes) generated and analysed during the current study are not publicly available due to maintaining confidentiality of the study participants but are available from the corresponding author on reasonable request.

## Abbreviations

HCW: Healthcare worker
HIV: Human immunodeficiency syndrome
MH: Municipal hospital
NTP: National tuberculosis control program
OPD: Outpatient department
PPE: Personal protective equipment
RHF: Rural health facility
SOP: Standard operating procedures
TSO: Task-shifting officer
WHO: World Health Organization
