# Supplementary material for: Barriers to tuberculosis case finding in primary and secondary health facilities in Ghana: perceptions, experiences and practices of healthcare workers
Source: BMC Health Serv Res. 2022 Mar 19;22:368. doi: 10.1186/s12913-022-07711-1 (PMC8934052; doi:10.1186/s12913-022-07711-1)
Supplement: Supplementary file 1 — Additional file 1. [file 12913_2022_7711_MOESM1_ESM.docx]

**Summary of checklist used for clinic observation**

| **Location within health facility** | **Observations done** |
| --- | --- |
| OPD waiting area | - Presence of TB posters - Whether health talks were held - Waiting time of clients - Behaviour of clients as they waited - Whether visibly coughing clients were separated from other clients - Communication style of HCWs and clients |
| Triage area | - Number of HCWs present - HCWs asking clients about cough and other TB related symptoms - Use of TB symptom screening tool - Number of clients who reported a cough - Recording of cough in a cough register - Requesting/referral for sputum test - Providing education on cough etiquette and how to produce sputum samples - escorting clients to the laboratory for sputum test or allowing them to go on their own - behaviour and communication style of HCWs and clients |
| Consulting room | - Number of HCWs present - HCWs asking clients about cough and other TB related symptoms - Use of TB symptom screening tool - Number of clients who reported a cough - Recording of cough in a cough register - Requesting/referral for sputum test - Providing education on cough etiquette and how to produce sputum sample - Escorting clients to the laboratory for sputum test or allowing them to go on their own - Behaviour and communication style of HCWs and client |
| Laboratory | - Number of HCWs present - Providing education on cough etiquette and how to produce sputum samples - Waiting time to submit sputum - Handling of sputum sample by client - How laboratory staff collected sputum from client - Behaviour and communication style of laboratory staff and clients |
| Chest clinic | - Number of HCWs present - Clients escorted to chest clinic by HCW or arriving alone - Providing education on cough etiquette - Counselling of clients with positive test results - Explaining treatment procedure to clients - Serving of medication to clients by HCWs - Behaviour and communication style of HCWs and client |
| General observations | - General facility layout and workflow - Whether any significant event was happening at the health facility on the day of observation |

HCW=healthcare worker, OPD=outpatient department, TB=tuberculosis
