# Supplementary material for: Barriers to tuberculosis case finding in primary and secondary health facilities in Ghana: perceptions, experiences and practices of healthcare workers
Source: BMC Health Serv Res. 2022 Mar 19;22:368. doi: 10.1186/s12913-022-07711-1 (PMC8934052; doi:10.1186/s12913-022-07711-1)
Supplement: Supplementary file 2 — Additional file 2. [file 12913_2022_7711_MOESM2_ESM.docx]

**Pathways to tuberculosis diagnosis and treatment in Ghana: identifying the gaps and seeking solutions**

**Health care worker qualitative semi-structured interview guide**

**General Information**

Interviewer Code: ______________ Note-taker Code: _____________

Date: ______/_______/__________ Start time: __________________

Name of Interviewer: ______________ Health facility: _______________

Description of place of interview:__________________________________________________________________________

**A. Demographic Characteristics**

1. Sex:

Male

Female

1. Profession: _________________________________________
2. Health Facility Unit: __________________________________

**B. Training in TB Control**

1. How long have you been working at this health facility? _______________________________
2. What role(s) do you play at this health facility? ______________________________________
3. What type of TB services are provided in your unit? (*probe for: education, diagnosis, laboratory, treatment, referral*) __________________________________________________
4. How long have you been involved with TB control at this health facility? __________________
5. Have you had any training on TB diagnosis and treatment? If yes, how many times have you been trained? ________________________________________________________________
6. How long ago was your last training on TB? _________________________________________

**C. Practices in TB diagnosis and treatment**

1. Do you have a cough register and how often are entries made in it?

*(look at a cough register with the HCW and discuss the gaps in the register (variables entered, last entry date etc)*

1. What do you do with the data entered in the cough register? ___________________________
2. If a patient reports a cough, what do you do? (*Probe: if HCW does not mention the use of a symptom screening questionnaire, then you ask about its use*).
3. How do you identify patients needing a TB test? (*Probe: for use of TB symptom screening questionnaire*)
4. If a patient is identified as requiring a sputum test, what do you do? *(Probe: education on sputum, lab request form, provide sputum container, refer to the TB lab*)
5. What do you do when a patient who has been asked to do a sputum test return with
   1. a positive test results? (*Probe: counselling, TB treatment*)
   2. a negative test results? (*Probe: counselling, normal treatment*)
6. Do you provide health education in your unit and does it include TB? *(Probe: how often health education is done (both group and individual education), content of TB it covers)*
7. What infection prevention and control measures do you take in this facility to prevent spread of TB *(Probe: promptly attending to patients with cough, promptly identifying and separating potential infectious TB patience)*
8. What guidelines do you follow in diagnosing TB in this health facility? ____________________
9. Do you have the guidelines available? ______________________________

(If yes, then look at the guidelines with the HCW *and probe reasons for aspects of the guideline that are implemented or not implemented, what is done at the waiting areas, OPD, consulting rooms, wards, laboratory and special clinics (ART, Diabetic, ANC, Hypertension Clinics*). But if No, show your guideline and go through the same procedure described above)

**D. Experience in TB case finding**

1. What will you say has been your experience with TB diagnosis and treatment in this health facility?

- What has been your experience with different categories/attitudes of patients (*Probe: age, sex, occupation, religion, tribe, location (rural, urban, country: Ghana, Togo))*.
- What has been your experience with screening for cough among patients visiting the health facility? (*Probe: recognition of symptoms, use of symptom questionnaire, referral from the consulting room*)
- What has been your experience with requesting for sputum test?
  - How easy is it for patient to do the test, receive results and initiate treatment for those with a positive test result? (*Probe: duration of sputum production, test duration, informing patient of results, patient initiation of treatment*)
  - What are the laboratory related factors that hinder requesting for sputum test? (*Probe: unavailability of sputum container, industrial strikes*)
- If a patient is diagnosed with TB and comes frequently to the facility, do you build a relationship with them *(Probe for the kind of relationship: friendly, cordial etc)*

**E. Barriers to TB diagnosis and treatment**

1. What in your view will you say are barriers to TB diagnosis and treatment in this health facility?

- In your opinion, what challenges do patients with TB symptoms face being diagnosed with TB and put on treatment in this facility? (*Probe: patient behaviour, human resource capacity at the facility, diagnostic capacity, logistics, infrastructure, program level challenges*)
- Are there any health worker challenges that undermine TB diagnosis? (*Probe: knowledge of TB, commitment/motivation, communication, risk of infection, stigma*)
- Away from the health facility, are people with TB symptoms facing challenges which make it harder for them to present at the facility? *(Probe: financial, social, religion, stigma)*. Is there anything that you do as a HCW about this?

**F. Suggested Solutions**

1. What do you think can be done to improve TB case finding in this facility?
2. Do you have any questions you will like to ask?

Thank you

Interview end time: ­­­­­­­­­­­­­­­­­­­­­­­___________________________
