# Supplementary material for: Barriers to tuberculosis case finding in primary and secondary health facilities in Ghana: perceptions, experiences and practices of healthcare workers
Source: BMC Health Serv Res. 2022 Mar 19;22:368. doi: 10.1186/s12913-022-07711-1 (PMC8934052; doi:10.1186/s12913-022-07711-1)
Supplement: Supplementary file 3 — Additional file 3. [file 12913_2022_7711_MOESM3_ESM.docx]

**Illustrative quotes on barriers to TB case detection**

| **Study results** | **Illustrative quotes** |
| --- | --- |
| **Health system-related barriers** | |
| Suboptimal screening for TB symptoms and  sputum test request | “Every client whether the person is a BP (blood pressure) client, […] because sometimes they easily forget something unless you remind them before they will say that oh yesterday, I was even coughing. So, we ask them”. (Male HCW, RHF) |
| Shortage of diagnostic materials | “[...] sometime sputum container will get finish, cartridge will get finish and sometimes what marvels me is that on days containers are in the lab they will be giving the containers out without checking their stock levels but will wait when I send a patient to them for sputum test before they will tell me that containers are finished. Sometimes containers will get finish and because the clients are coming plenty and everybody is at risk, so I have to go and […] have to give me some money to go and look for containers […]”. (Male HCW, MH) |
| Poor documentation in cough  registers | “We don’t have enough staff to record cough in the register. Actually the one who was doing this is no longer in the facility”. (Female HCW, RHF) |
| Sub-optimal infection  prevention and control practices | “Here the relatives can enter anytime they want, maybe in the night that I don’t know but for daytime the relatives enter the isolation ward anytime they want […], that’s one of our challenges in this premises, the regulation is not there”. (Male HCW, MH) |
| Insufficient monitoring and supervision of TB work by TB  team | “There is a name to that effect, yes in quote a TB Team, but we don’t conduct any meetings I mean nothing really happens so I really don’t know if that team is functional or not but it’s just a name [..]”. (Male HCW, MH) |
| **Healthcare worker-related barriers** | |
| Gaps in TB knowledge and lack of training in case detection guidelines | “The nurses at the hospital here we are many and if all of us we know something about tuberculosis and about tuberculosis detection, if all of us we are aware and we know much about it, we will be able to identify more cases but because most of us don’t know anything about tuberculosis when a person is even coughing they will ignore the person until those experience ones capture the person”. (Male HCW, MH) |
|  | “Not all the health workers have gone for the training, but TB is a common thing [..], we have been learning about it so even if you don’t go for training at least you have to identify cases”. (Female HCW, RHF) |
| Fear of infection | “If the health worker doesn’t have so much knowledge about TB and has just this knowledge that TB is infectious, […] then getting closer to the person and even how to handle yourself before the person so that you will not also get infected is one issue.  Also, if the nurse doesn’t have much knowledge then stigmatization then comes in and fear of infection comes in too so you will not be able to assess the client very well”. (Female HCW, RHF) |
| Attitude towards TB work | “Some of our laboratory staff, who show much concern for this screening issue, sometimes if accidentally they are not there and those who are around are the less concerned ones, sometimes we don’t get test result. [Therefore we] sometimes have to recall the clients to come and reproduce the sputum, sometimes you have to pay  their T&T (travel and transportation)”. (Male HCW, MH) |
| Suggested solutions by HCWs | “If we get laboratory in any of the four facilities in the sub-district, it will solve the referral problem”. (Female HCW, RHF) |
|  | “[…] instead of we referring directly to the laboratory at the municipal hospital, there could be a focal person at the laboratory who could come and take the sample and then when the person is diagnosed positive, the person can now go to the  bigger facility for treatment […]”. (Female HCW, RHF) |

HCW= healthcare worker, MH= municipal hospital, RHF= rural health facility, TB= tuberculosis
